# Diagnostic and Treatment Challenges of Adult-Onset Still’s Disease in the Background of Pulmonary Tuberculosis

**DOI:** 10.7759/cureus.70287

**Published:** 2024-09-26

**Authors:** Ajay Kurmi, Sandipa Sharma, Japana Regmi, Sheekha Pokhrel, Sanjeela Gurung

**Affiliations:** 1 Department of Nephrology, Shahid Dharma Bhakta National Transplant Centre, Bhaktapur, NPL; 2 Department of Medicine, Shivanagar Primary Health Care Center, Bharatpur, NPL; 3 Department of Medicine, College of Medical Sciences, Bharatpur, NPL; 4 Department of Medicine, Nepal Medical College, Kathmandu, NPL; 5 Department of Medicine, Lumbini Medical College and Teaching Hospital, Palpa, NPL

**Keywords:** adult-onset still’s disease, antitubercular therapy, arthralgia, evanescent skin rash, pulmonary tuberculosis

## Abstract

Adult-onset Still's disease is a multisystem inflammatory disease characterized by fever, rashes, arthritis, and sore throat. The occurrence of this disease and pulmonary tuberculosis is infrequent, and more data are needed in the literature regarding both conditions. We present the case of a 48-year-old woman undergoing treatment for sputum-positive pulmonary tuberculosis who presented with features suggestive of adult-onset Still’s disease. A detailed evaluation led to the diagnosis of adult-onset still’s disease with pulmonary tuberculosis, and the patient was managed accordingly.

## Introduction

Adult-onset Still's disease (AOSD) is a rather uncommon multisystem inflammatory disorder in which patients present with features such as high spiking fever, sore throat, polyarthritis, and an evanescent salmon-colored rash during the height of the fever [1]. Although the etiology of AOSD remains unknown, it is one of the causes of pyrexia of unknown origin and is a diagnosis of exclusion, as the symptoms overlap with multiple other diseases [2]. With a prevalence of one in 100,000, AOSD is an uncommon disease worldwide [3]. Although definite etiology is unknown, its predilection is seen slightly more in females than males. A bimodal age distribution has also been observed with the first peak between the ages of 15 and 25 and the second peak between 36 and 46 years [4].

Although tuberculosis is a highly prevalent disease, its occurrence with AOSD is rare. Here, we report an unusual case of AOSD in a 48-year-old woman who had a month-long history of diagnosed sputum-positive pulmonary tuberculosis (PTB) undergoing antitubercular therapy. She presented to our center with additional symptoms of high-grade fever and arthralgia along with persistent abnormalities in blood cell counts. Differential diagnoses at presentation included tuberculous bronchopneumonia, pyrazinamide-induced allergic reaction and hyperuricemia, hematological malignancies, and autoimmune disorders.

## Case presentation

A 48-year-old woman presented with complaints of easy fatigability and fever for four months. She had an intermittent spiking fever associated with chills and rigors, especially in the evening with a maximum temperature recorded as 103°F. Additionally, she reported malaise and body aches for one month associated with bilateral symmetrical multiple joint pain and morning stiffness. Her symptoms were temporarily relieved with acetaminophen. Further history revealed a pink-colored rash occurring at the onset of fever and involving the neck, upper limb, and abdomen for four days. She was diagnosed with smear-positive PTB (rifampicin-sensitive) one month ago at a different health center, for which she was taking antitubercular therapy. Moreover, her history revealed persistent anemia, leukocytosis, and thrombocytosis, for which multiple investigations were done. Blood and urine investigations for infectious etiology, including blood and urine cultures, Brucella antigen, and scrub typhus antibody, were negative. No definite cause was identified to explain her clinical features and lab workups. However, her previous records showed she was treated with amoxicillin-clavulanic acid, levofloxacin, and doxycycline from multiple centers during this period.

Examination revealed a well-built female with stable vitals. On head-to-toe examination, pallor and bilateral knee joint swelling were seen. Rashes could not be appreciated during the examination. Bilateral basal crepitation was heard during pulmonary examination, while other systemic examinations were within normal limits.

**Figure 1:**
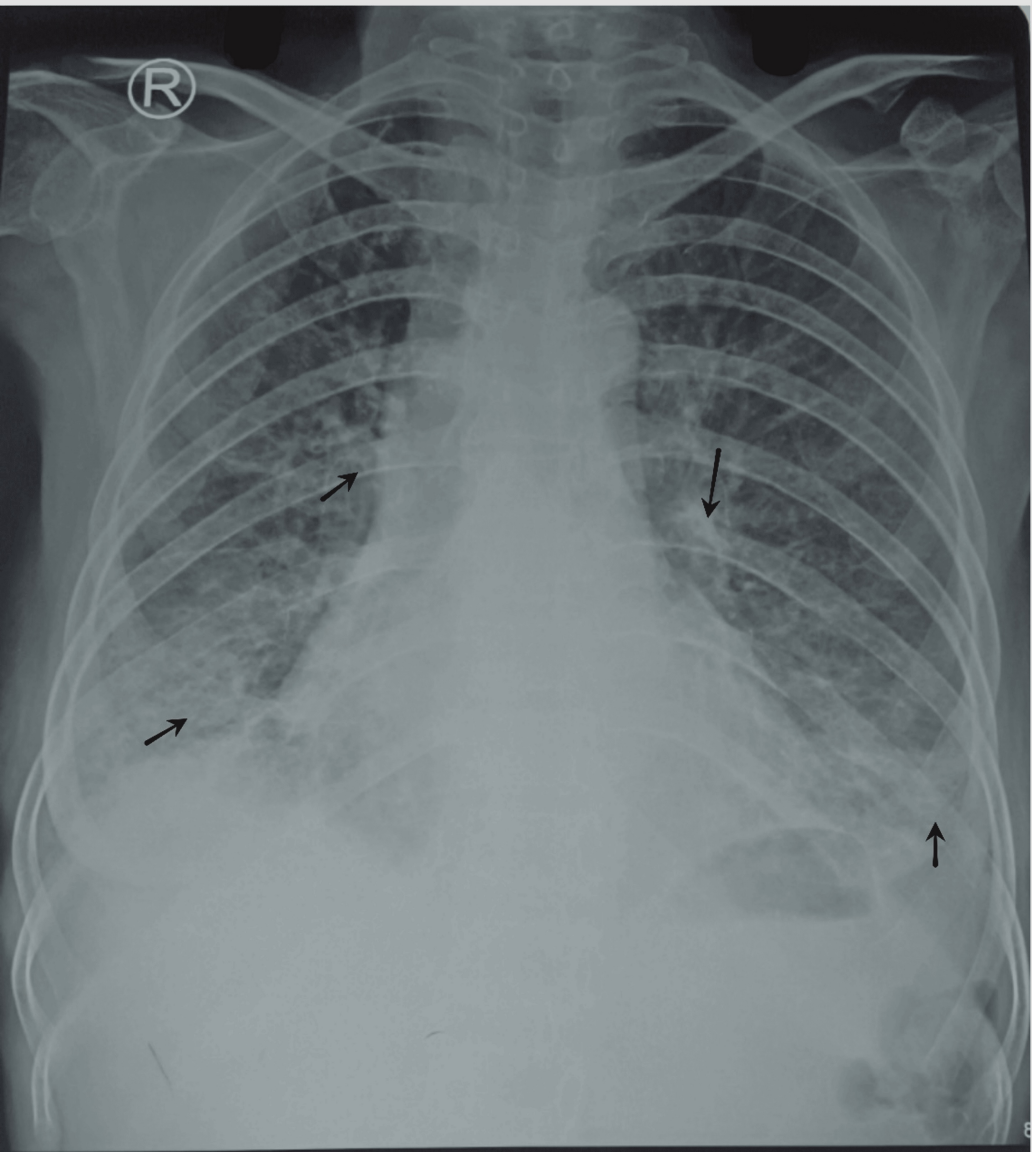
Chest X-ray of the patient showing bilateral hilar fullness and bilateral lower zone haziness.

Laboratory studies revealed markedly elevated serum ferritin levels with decreased serum total albumin levels. Complete blood counts showed decreased hemoglobin and increased leukocyte and platelet counts, as in her blood tests from a month ago. Other tests for renal function and coagulation profile were normal. Serological tests for syphilis, hepatitis, human immunodeficiency virus (HIV), antinuclear antibody, anti-cyclic citrullinated peptide antibody, and rheumatoid factor were also negative. Chest X-ray revealed findings suggestive of ongoing pulmonary infection, most likely due to PTB. Further investigations, such as computed tomography (CT) scan of the chest or bronchoscopy, could not be performed due to the patient's refusal, citing the high cost. Figure 1 shows the chest X-ray, and Table 1 shows detailed laboratory findings.

**Table 1:** Laboratory examination of the patient. TLC: total leukocyte count; ESR: erythrocyte sedimentation rate; CRP: C-reactive protein; ALT: alanine transaminase; AST: aspartate transaminase; Ab: antibody.

Parameters	December 1, 2023	January 3, 2024	Reference range
Hemoglobin (gm/dL)	8.6	9.2	11-16
TLC (/mm^3^)	24,400	12,000	4,000-11,000
Platelet count (/mm^3^)	492,000	591,000	150,000-400,000
ESR (mm/hour)	60	61	5-15
CRP (mg/dL)	40	48	0.3-1.0
Serum albumin (gm/dL)		1.91	3.5-5.0
ALT (U/L)		60	5-25
AST (U/L)		146	8-40
Ferritin (μg/L)		579.91	15-150
Brucella antigen	Negative		
Scrub typhus Ab	Negative		

Two sputum smear samples were tested, which were negative for acid-fast bacilli (AFB). A bone marrow biopsy revealed normocellular marrow with megaloblastic maturation but was negative for hematological malignancies. After ruling out all other possible diagnoses and based on Yamaguchi's criteria, she was diagnosed with AOSD with PTB. The patient was started on a daily dose of 40 mg oral prednisolone, which was decreased by 10 mg each week until 10 mg. Subsequently, she was maintained on 5 mg prednisolone for two months and then discontinued. Due to its anti-inflammatory properties, naproxen was added to the treatment for a week. Antitubercular therapy was continued. She was advised on weekly follow-up and treated on an outpatient basis until her symptoms subsided. On follow-up a month later, her symptoms subsided, laboratory values were improving, and she was able to resume her daily activities.

## Discussion

The specific etiology is unknown; however, in genetically predisposed individuals, it is likely associated with an autoinflammatory cascade due to an abnormality in the innate immune system, and involvement of cytokines such as interleukin (IL)-1, IL-6, IL-18, and tumor necrosis factor (TNF)-alpha is believed to play a role [5]. Furthermore, the inflammatory cascade in AOSD is found to have been triggered by specific infections, solid cancers, and hematological malignancies [6]. The inflammatory cascade in our patient was probably in the background of PTB.

Laboratory examinations typically show leukocytosis with neutrophilia, thrombocytosis, anemia, and an elevation in inflammatory markers such as erythrocyte sedimentation rate (ESR), C-reactive protein (CRP), and serum ferritin. In our patient, detailed investigations were conducted after her tests revealed persistent hematological abnormalities as described above.

It has been seen that AOSD is difficult to diagnose, as it is a rare disease that mimics symptoms of other diseases, including systemic infections (such as viral infections, HIV, parvovirus B19, viral hepatitis, measles, and toxoplasmosis), malignancies (malignant lymphomas, leukemia, myeloproliferative disorders, and solid organ tumors), autoimmune disorders (such as systemic lupus erythematosus and rheumatoid arthritis), and autoinflammatory diseases (such as familial Mediterranean fever), which makes it a diagnosis of exclusion [7,8]. Given the history of rash and arthritis, the clinical presentation of our patient also resembles features of pyrazinamide-induced allergic reaction and hyperuricemia. However, the drug-induced allergic rash is usually bright red and pruritic [9]. The rash in our patient was pink, non-pruritic, and evanescent, which points more toward AOSD. Moreover, her affected joints were non-tender and without erythema (as expected in gouty arthritis), which rules out drug-induced gout [10]. Several criteria have been proposed to aid the diagnosis, with the Yamaguchi criteria being the most commonly used. Five or more criteria are required for the diagnosis, among which two or more criteria must be major. Diagnosis is excluded in patients with exclusion criteria [11]. Our patient met the criteria with four major and two minor criteria being positive. Also, various blood tests, including cultures, serology, and bone marrow biopsy, were negative for infections, malignancies, and rheumatological diseases. Table 2 shows Yamaguchi’s criteria for the diagnosis of AOSD.

**Table 2:** Yamaguchi’s criteria. Source: Wong-Pack et al. [7].

Major criteria	Minor criteria	Exclusion criteria
1) Fever ≥39°C persisting for ≥1 week	1) Sore throat	1) Infections, in particular sepsis and infectious mononucleosis
2) Arthralgia/arthritis persisting for ≥2 weeks	2) Lymphadenopathy and/or splenomegaly	2) Malignancy, in particular lymphoma
3) Typical rash	3) Increased serum aminotransferase or lactate dehydrogenase levels (after other causes have been excluded)	3) Other rheumatic diseases, in particular polyarteritis nodosa and vasculitis in the course of rheumatoid arthritis
4) White blood cell count ≥10×10^9^/L (>80% neutrophils)	4) Negative IgM rheumatoid factor and antinuclear antibodies (immunofluorescence assay)	

The treatment of AOSD is guided by the disease's pattern and the patient's clinical manifestation [12]. Therapeutic options include non-steroidal anti-inflammatory drugs, glucocorticoids, disease-modifying antirheumatic drugs, IL-1 inhibitors, and IL-6 inhibitors [13]. It was a challenging scenario in the treatment of our patient, as she was already diagnosed with PTB. A similar case has been published, which emphasizes the role of colchicine in the treatment of patients diagnosed with AOSD and with contraindications for treatment with glucocorticoids and other immunosuppressants [14]. However, there is limited literature available on this treatment to date. In our patient, with two negative sputum samples for AFB, glucocorticoids were started with close monitoring for symptoms and repeated sputum smear examination. Subsequent sputum smears came negative for AFB, and the patient also reported resolving symptoms.

## Conclusions

AOSD is rare. With clinical features mimicking various systemic diseases and having no specific diagnostic tests, its diagnosis is challenging. Further, the clinical symptoms and their chronicity also led to other differential diagnoses such as malignancies and rheumatic diseases. This brings more challenges to physicians when complicated by the coexistence of diseases like tuberculosis. Therefore, one must be vigilant regarding the coexistence of AOSD and other possible diagnoses, whether infectious or malignant. Moreover, if glucocorticoids or immunomodulators cannot be used either due to contraindications or affordability, focus should be made on starting alternative drugs or using those drugs carefully.
